# Dilemma in Differentiating between Acute Osteomyelitis and Bone Infarction in Children with Sickle Cell Disease: The Role of Ultrasound

**DOI:** 10.1371/journal.pone.0065001

**Published:** 2013-06-06

**Authors:** Baba P. D. Inusa, Adeola Oyewo, Felicity Brokke, Gayathriy Santhikumaran, K. Haran Jogeesvaran

**Affiliations:** 1 Department of Paediatrics, Evelina Children’s Hospital, Guy’s and St. Thomas’ National Health Service (NHS) Foundation Trust, London, United Kingdom; 2 King’s College London School of Medicine, London, United Kingdom; 3 Department of Paediatric Radiology, Evelina Children’s Hospital, Guy’s and St. Thomas’ NHS Foundation Trust, London, United Kingdom; Indian Institute of Science, India

## Abstract

**Background:**

Distinguishing between acute presentations of osteomyelitis (OM) and vaso-occlusive crisis (VOC) bone infarction in children with sickle cell disease (SCD) remains challenging for clinicians, particularly in culture-negative cases. We examined the combined role of ultrasound scan (USS), C - reactive protein and White blood counts (WCC) in aiding early diagnosis in children with SCD presenting acutely with non-specific symptoms such as bone pain, fever or swelling which are common in acute osteomyelitis or VOC.

**Methods:**

We reviewed the records of all children with SCD who were discharged from our department from October 2003 to December 2010 with a diagnosis of osteomyelitis based on clinical features and the results of radiological and laboratory investigations. A case control group with VOC who were investigated for OM were identified over the same period.

**Results:**

In the osteomyelitis group, USS finding of periosteal elevation and/or fluid collection was reported in 76% cases with the first scan (day 0–6). Overall 84% were diagnosed with USS (initial +repeat). 16% had negative USS. With VOC group, USS showed no evidence of fluid collection in 53/58 admissions (91%), none of the repeated USS showed any fluid collection. Mean C-reactive protein (CRP), and white cell count (WCC) were significantly higher in the OM.

**Conclusion:**

The use of Ultrasound in combination with CRP and WCC is a reliable, cost-effective diagnostic tool for differentiating osteomyelitis from VOC bone infarction in SCD. A repeat ultrasound and/or magnetic resonance imaging (MRI) scan may be is necessary to confirm the diagnosis.

## Introduction

Sickle Cell Disease (SCD) is a clinically significant haemoglobinopathy with increasing incidence in developed countries. SCD affects 1 in every 2000 births in England and it is estimated that there are about 12–15,000 SCD patients in the UK [1]. However this pales in significance when compared to Sub-Saharan Africa where over 75% of the world’s patients live. The underlying pathology is the obstruction of the microvasculature by sickled red blood cells, resulting in chronic tissue ischaemia and tissue infarction; this may present as pain and/or swelling referred to as vaso-occlusive crisis (VOC). Vaso-occlusive events are the most common acute clinical presentation of SCD in children [2]. A combination of tissue infarction, immunodeficiency due to splenic dysfunction and excess iron leads to increase risk of osteomyelitis in SCD. Salmonella osteomyelitis is several hundred times more likely to occur in children with SCD than in the general population [2]–[4].

In the acute stage, VOC and osteomyelitis are almost indistinguishable. Both conditions are associated with a rise in inflammation such as C-reactive protein (CRP) and white cell count (WCC). Although VOC bone infarction is reported to be up to 50 times more common than osteomyelitis in patients with SCD [5], [6]. To forestall the development further complication, it is important to establish the correct diagnosis. The gold standard for diagnosing osteomyelitis is a positive blood culture or joint aspirate, but a negative blood culture does not exclude the diagnosis of osteomyelitis (4–6).

Magnetic Resonance Imaging (MRI) is a useful tool in diagnosing osteomyelitis in patients with SCD [6], [7]; especially with contrast enhancement [7]–[9]. MRI scans are not always readily accessible; this may be due either to cost or availability. However ultrasound scans (USS) alone has previously been shown to be a useful investigation in diagnosing osteomyelitis in patients with SCD with sensitivity of 74% and a specificity of 63% [10], [11]. The aim of this study was to assess the additional benefit of CRP and WCC as a tool in aiding early diagnosis in children with SCD presenting acutely with bone pain, fever or swelling.

## Methods

### Ethics Statement

This study was reviewed by the Specialty Clinical Audit Directorate at the Trust and approved by the Clinical Governance department. This study was approved by the clinical audit committee as part of clinical governance following a peer review process. Any data for publication must be anonymised. This document has no patient identifiers in whatever form. According to the Governance Arrangements for Research Ethics Committees (GAfREC, 2005) this study does not require IRB review.

### Recruitment

**Figure 1:**
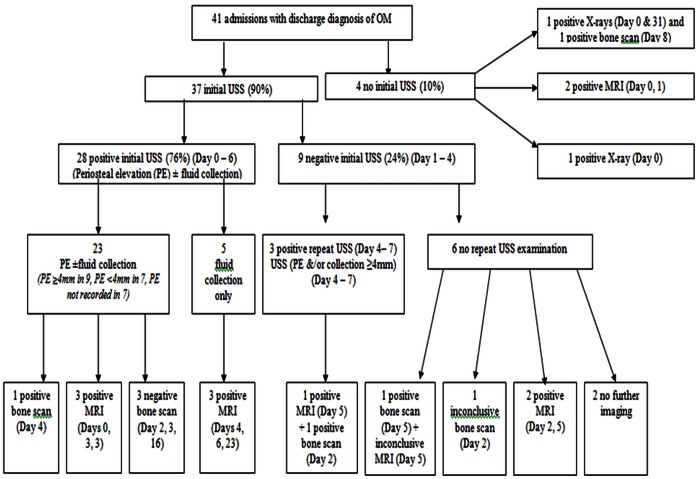
The results of imaging investigations in the osteomyelitis (OM) cohort.

**Figure 2:**
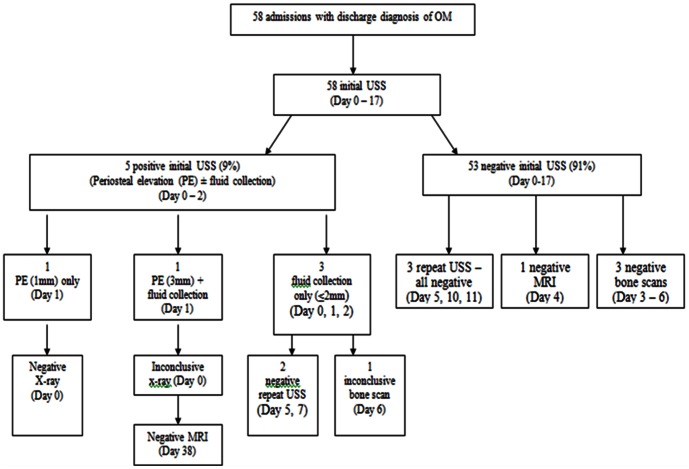
The results of imaging investigations in the vaso-occlusive crisis (VOC) cohort.

**Figure 3:**
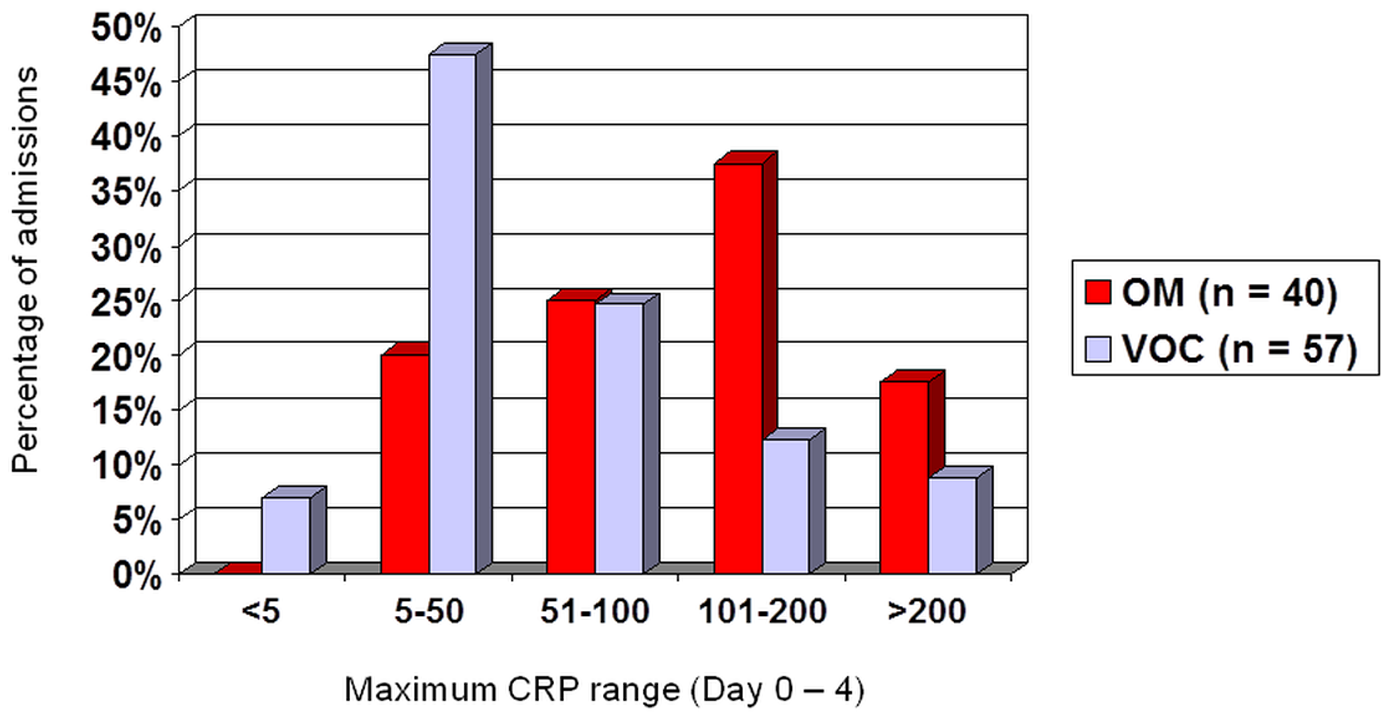
Maximum CRP range (day 0–4) in the osteomyelitis (OM) and vaso-occlusive crisis (VOC) group.

The radiology information system (Clinical Research Information System, CRIS) was interrogated to identify all paediatric patients aged 0 to 18 years age who were admitted to our department over a period of 7 years and 2 months (October 2003 to December 2010) with suspected osteomyelitis or VOC and underwent imaging studies as part of the diagnostic work-up see Figure 1 & 2. This information was cross-checked with the SCD database. Chronic OM was excluded. Patient case notes and Electronic Patient Records (EPR) were examined to collect data including symptoms at presentation and results of laboratory investigations including WCC), CRP (Figure 3), blood culture results and bone or joint aspirate results. Findings from surgical interventions where applicable, were also recorded.

### Osteomyelitis (OM) Cohort

**Figure 4:**
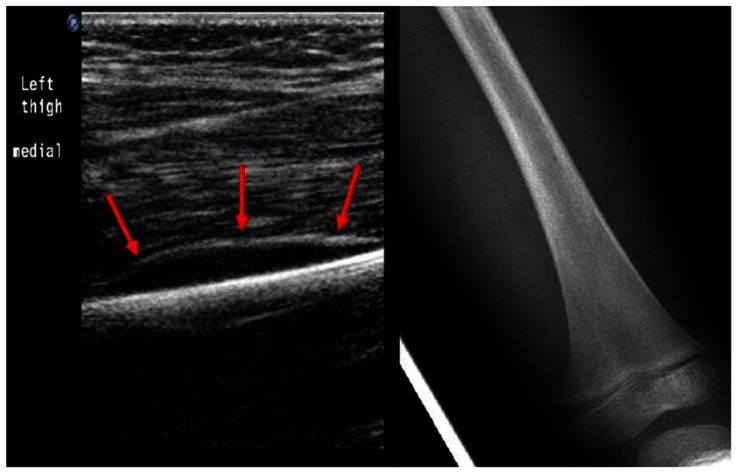
Lower limb USS I a 3-year-old patient with osteomyelitis.

**Figure 5:**
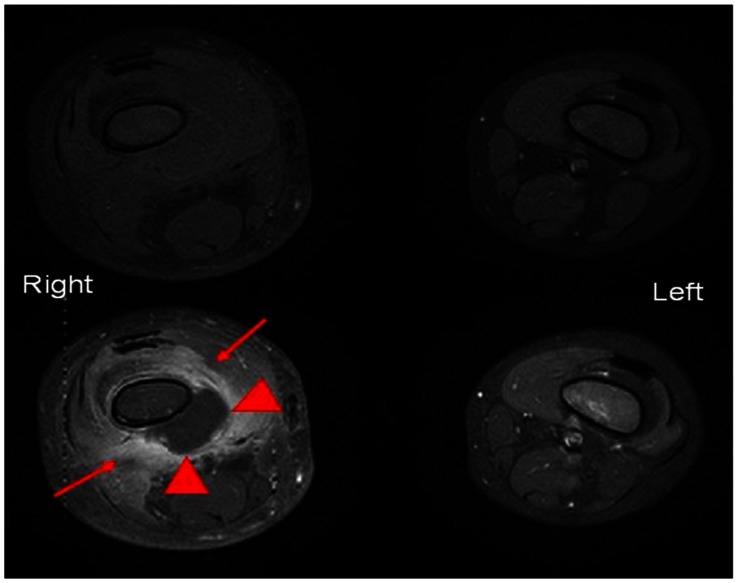
Axial MRI images through the distal femora in a 4-year-old with osteomyelitis.

A total of forty one (41) SCD patients were included in the review with a discharge diagnosis of osteomyelitis.. The discharge diagnosis of osteomyelitis was based on positive radiological findings interpreted together with haematological investigations, blood cultures, and bone or joint aspirate. A positive USS finding for diagnosis of osteomyelitis was defined as presence of a significant periosteal elevation (>0.4 cm) in accordance with (10,11) as shown in Figures 4. All but four US images were review by HJ. The MRI scan (figure 5) and nuclear medicine bone scan was considered to be positive if it was reported by the consultant radiologist as showing changes consistent with osteomyelitis in line with Jain et al, 2008 (7).

### Vaso-occlusive Crisis (VOC) Cohort

We included a total of fifty eight SCD patients (table 1) who presented within the study period with features suggestive of OM but had a discharge diagnosis of VOC based on negative blood cultures, radiological findings. Thirty-four patients in this group with a total of fifty-eight admissions; thirteen patients had two or more admissions.

**Table 1:** Background data of admissions in osteomyelitis (OM) and vaso-occlusive crisis (VOC) groups.

Characteristics		OM (*n* = 41)	VOC Group (*n* = 58 )
Male *(%)*		31 *(76)*	37 *(64)*
Female *(%)*		10 *(24)*	21 *(36)*
Age, years, *M*, *(SD)*		5.8 *(5)*	5.9 *(4)* †
Age ranges *(%)*	<2	8 *(20)*	5 *(9)* †
	2–4	14 *(34)*	21 *(36)* †
	5–9	10 *(24)*	22 *(38)* †
	>9	9 *(22)*	10 *(17)* †
SCD genotype, No. *(%)*			
HbSS		34 *(83)*	48 *(83)* †
HbSC		7 *(17)*	8 *(14)* †
HbS/β-thalassemia		0	2 *(3)* †
PMH of OM, No *(%)* *		11/36 (31)*	9/50 (18)*

† not statistically significant, p>0.05.

* The number and percentage of admissions that were recorded as ‘yes’.

### Statistical Analysis

An independent-samples t-test was conducted to compare variables from the osteomyelitis group and VOC group such as duration of symptoms prior to admission and presence of periosteal elevation or fluid collection on USS. Mean CRP and WCC at presentation as well as maximum CRP from day 0 to day 4 of admission were compared in the osteomyelitis and VOC group using independent-samples t-test. Statistical analyses were performed using Statistical Package for the Social Sciences (SPSS/PASW, version 18.0, Chicago, IL, USA) and differences with a p value<0.05 were considered statistically significant.

## Results

Background demographic data including the symptoms at presentation and duration of these symptoms in osteomyelitis and vaso-occlusive crisis cohorts are illustrated in Tables 1, 2, 3 below. Initial and repeat USS findings in both cohorts are shown in Tables 4 and 5. The level of inflammatory marker response shown between the two groups on Table 6.

**Table 2:** Symptoms at Presentation in osteomyelitis (OM) and vaso-occlusive crisis (VOC) groups.

Symptoms	OM Group(*n* = 41)	VOC Group(*n* = 58 )
Pain, (%)	40 *(98)*	*58 (100)*
Fever >38°C, (%)	16 *(39)*	*25 (43)*
Reduced range of movement, (%)	16 *(39)*	29 *(50)*
Swelling (%)	33 *(81)*	38 *(66)*

**Table 3:** Duration of Symptoms in osteomyelitis (OM) and vaso-occlusive crisis (VOC) groups.

Duration of Symptoms	OM Group(*n* = 41)	VOC Group(*n* = 58 )
Minimum duration (days)	1	1
Maximum duration (days)	30	20
Mean duration (SD) (days)	5.6 (5.5)	4.7 (3.4)

**Table 4:** Initial USS findings in osteomyelitis (OM) and vaso-occlusive crisis (VOC) groups.

Initial USS Findings	OM Group (n = 37)*	VOC Group *(n = 58)*
Positive USS *(%)*	28 (76)	5 (9)
Periosteal elevation (PE) only *(%)*	12 (32)	1 (2)
Fluid collection only *(%)*	5 (14)	3 (6)
PE+Fluid collection *(%)*	11 (30)	1 (2)
Negative USS *(%)*	9 (24)	53 (91)
Mean no. of days after presentation *(SD*)	1.7 (1.5)	2.2 (2.5)

Note: *USS data for 4/41 admissions in the osteomyelitis group could not be obtained, but reports from other imaging studies (X-ray, nuclear medicine bone scan and MRI) confirmed their diagnosis.

**Table 5:** Repeat USS findings in osteomyelitis (OM) and vaso-occlusive crisis (VOC) groups.

Repeat USS Findings	OM *(n = 16)*	VOC Group *(n = 4)*
Positive repeat USS (%)	13 (81)	0 (0)
Periosteal elevation (PE) only *(%)*	5 (31)	0 (0)
Fluid collection only *(%)*	2 (13)	0 (0)
PE+Fluid collection *(%)*	6 (38)	0 (0)
Negative repeat USS (%)	3 (18.75)	4(100)
Mean no. of days afterpresentation *(SD*)	6.9 (5.0)	8.3 (2.8)

**Table 6:** Initial C-reactive protein (CRP), white cell count (WCC) and maximum CRP values in osteomyelitis (OM) and vaso-occlusive crisis (VOC) groups.

	OM group (n = 41)	VOC group (n = 58)
Initial CRP≥5 (%)	34 (83)	42 (72.41%)
Initial CRP<5 (%)	4 (10)	10 (17)
Initial CRP Not recorded (%)	3 (7)	6 (10)
Mean Initial CRP (SD)	86.4 (66.2) †	39.8 (52.4) †
*Mean Maximum CRP (SD)	119.2 (73.6) ††	70.5 (71.2) ††
Initial WCC >11 (%)	36 (88)	39 (67)
Initial WCC ≤11 (%)	4 (10)	18 (31)
Not recorded (%)	1 (2)	1 (2)
Mean Initial WCC (SD)	17.8 (6.5) †††	14.9 (6.6) †††

† p = 0.001, two-tailed,

†† p = 0.002, two-tailed,

††† p = 0.046, two-tailed.

(p<0.05 =  statistically significant).

* Mean of maximum CRP recorded day 0–4 of admission.

**Osteomyelitis (OM) Group: -** total number of admissions of patients with Sickle Cell Disease (SCD) and Osteomyelitis (OM).

**Vaso-occlusive crisis (VOC) Group: -** total number of admissions of patients with SCD and vaso-occlusive crises (VOC).

### Osteomyelitis Cohort

Figure 1 illustrates the results of imaging investigations in the osteomyelitis group, a positive USS was reported in twenty-eight (76%) of the thirty-seven investigations on admissions(≤6) and a repeat USS between days 4–7 of presentation, were found to have sub-periosteal fluid collections ≥4 mm in depth. Therefore USS was diagnostic in 84%. The remaining six (16%) with negative USS in osteomyelitis group required MRI, to confirm the diagnosis. Eight of the forty-one admissions in this cohort had a triple phase bone scan on days 2–16 of admission but this was not contributory to final diagnostic category. Mean CRP at presentation 86.4±66.2 and Mean WCC 17.8±6.5 (Table 6). Blood culture was taken in thirty-nine of the forty-one admissions, of these, two (5%) had positive blood culture results. One sample grew *Morganella morganii* – a gram negative bacteria and the other grew *mycoplasma pneumoniae*. A total of eighteen of the forty-one admissions had surgical incision and drainage at the affected joint, of the sixteen specimens, three grew *Salmonella spp*.

### Vaso-occlusive Crisis (VOC) Cohort

Figure 2 illustrates the results of imaging investigations undertaken in VOC cohort. Five of the fifty-eight admissions had initial sub-periosteal fluid collections on ultrasound. Of these, small fluid collections ≤2 mm diameter, were noted in three admissions (5%) only. The remaining two (3%) admissions with periosteal fluid 1–3 mm USS. Overall, in this cohort, two cases had MRI scans (4 and 38), both were reported as infarcts. Mean CRP 39.8±52.4 (Table 6) and Mean initial WCC 14.9±6.6). Blood cultures were taken in forty-nine of the fifty-eight admissions, all samples were sterile.

### Comparing Initial USS Diagnostic Findings and Haematological Markers of Infection in OM and VOC Groups

The number of days from start of symptoms until presentation was not statistically significant for the osteomyelitis and VOC groups.

There was a significant difference in detection of periosteal elevation [(*t* (41) = −7.1, *p*<0.0005 (two-tailed)] and fluid collection [(*t* (49) = −3.3, ***p =*** **0.002** (two-tailed)] for the osteomyelitis group and VOC groups.

There was a significant difference in mean WCC at presentation for the osteomyelitis group (*M* = 17.8, *SD* = 6.5) and VOC group (*M* = 14.9, *SD* = 6.4; *t* (86) = 2.0, ***p =*** **0.046, two-tailed)**. There was also a significant difference in mean CRP at presentation for the osteomyelitis group (*M* = 86.4, *SD* = 66.2) and VOC group (*M* = 39.8, *SD* = 52.4; *t* (63) = 3.5, *p = *0.001, two-tailed). Mean maximum CRP for OM = 119.2, *SD* = 73.6 and VOC = 70.5, *SD* = 71.2; *t* (91) = 3.2, *p = *0.002, two-tailed).

Our findings indicate that significant elevation in CRP and WCC in the presence of bone swelling and fever should trigger USS as a first line test. This also validates the fact that periosteal elevation and/or collection of fluid on USS in children with SCD is highly suggestive of osteomyelitis even in the absence of positive blood cultures.

## Discussion

Despite recent advances in diagnostic investigations, distinguishing acute presentation of OM from VOC in culture-negative patients remains a clinical conundrum. Various imaging modalities have been employed in aiding the diagnosis of osteomyelitis each with its benefits and disadvantages [12]–[14].

USS has been reported to have high sensitivity in diagnosing osteomyelitis in children with SCD [10], [11]. Our findings of 76% initial USS sensitivity are consistent with those previously reported [10]. We had an initial false negative rate of 24% which were later confirmed with a repeat USS,/MRI in the presence of high WCC and/or CRP. Williams et al. (2000) [10] reported that USS finding of ≥4 mm of sub-periosteal fluid collection is a strong indicator of osteomyelitis in admissions with SCD. 32% of admissions in our osteomyelitis group had periosteal elevation or fluid collection ≥4 mm. Five of the fifty-eight admissions in the VOC group had periosteal elevation even though <4 mm on initial USS, giving an initial false positive rate of about 9%, repeat USS findings were all negative in this cohort.

Although contrast enhanced MRI have been shown to allow improved definition of the site and extent of bone involvement in osteomyelitis [6], [8]; its ability to accurately distinguish between acute presentations of osteomyelitis and infarction in children with SCD is subject to debate. Umans et al (2000) [8] reported that contrast-enhanced MRI could allow accurate distinction between acute infarct and osteomyelitis however this finding was contradicted by Bonnerot et al. (1994) [9] who concluded that enhanced MRI could not reliably distinguish infarction from osteomyelitis. MRI findings were reported as being consistent with osteomyelitis in eleven of the twelve admissions in our osteomyelitis cohort who had MRI scans, one report was inconclusive.

The use of bone scans in differentiating between acute osteomyelitis and VOC has been reported [12]–[13]. Nuclear medicine scans were not contributory in our series which is consistent with previous reports [6], [14]. In our study, eight admissions in the osteomyelitis had bone scans, only two of which were consistent with the final diagnosis.

### Limitations

This study has limitations; its retrospective nature meant difficulty was encountered in data collection; six patients were excluded due to insufficient data. Not all admissions with VOC underwent imaging therefore by selecting patients who had imaging as part of their initial work up we may have introduced selection bias towards more severe cases of VOC. One patient in the VOC group was found to have periosteal elevation of 3 mm and fluid collection on initial USS on day 1 of admission, MRI on day 38 revealed a mature infarct in the affected joint in this patient. At the time, a diagnosis of VOC was given and the patient responded to treatment with fluids and analgesia according to the VOC protocol. Although WCC was within normal limits and CRP was only slightly raised at 7.0, in this patient, it is possible that this could have been a case of sub acute osteomyelitis superimposed on an infarct.

### Conclusion

Based on our finding the use of ultrasound for diagnosis of OM is enhanced when taken in combination with the CRP/WCC level on admission. The choice and duration of antibiotic treatment will be guided by the final diagnosis. We suggest a significant elevation of CRP and WCC even in absence of periosteal fluid elevation on initial USS may warrant a repeat USS study or MRI scan to exclude OM.
